# The experiences of sexual and gender minorities accessing perinatal care: A scoping review

**DOI:** 10.1177/17455057261444891

**Published:** 2026-05-10

**Authors:** Billie Zeta, Tejaswini Patil

**Affiliations:** 1 Department of Social Work, Monash University, Melbourne, VIC, Australia

**Keywords:** LGBTIQA+, pregnancy, health equity, queer, perinatal

## Abstract

**Background:**

Sexual and gender minorities (SGMs) experience discrimination and stigma when accessing healthcare due to the prevalence of dominant heterocisnormative culture, which leads to poorer health outcomes when compared to the general population. Current international perinatal care policies mostly do not acknowledge the existence of SGMs or their health care needs.

**Objectives:**

To conduct a scoping review that examines the existing international literature on the experiences of childbearing SGMs accessing perinatal care.

**Eligibility Criteria:**

English-language primary evidence discussing the experiences of SGMs accessing perinatal care. Eligibility was not restricted by country or study design.

**Sources of Evidence:**

Studies were sourced from CINAHL, Scopus and OVID Medline using standardised search strategies.

**Chartings Methods:**

Data were charted in accordance with the guidelines and recommendations outlined by the Joanna Briggs Institute Evidence Synthesis and the Preferred Reporting Items for Systematic Reviews extension for Scoping Reviews (PRISMA-ScR).

**Results:**

Sixteen studies were identified as eligible for inclusion. Studies originated from predominantly western countries and contexts. Participants were predominantly white and highly educated where demographic data were available. Five themes were identified: structural stigma, interpersonal stigma, affirmation of identity, resilience and positive experiences. Intersex experiences are under-researched.

**Conclusion:**

The findings demonstrate several barriers and facilitators experienced by SGMs while accessing perinatal care. Policies should be developed to address stigma and barriers and support inclusive practice, alleviating the burden on SGM people to resist heterocisnormativity when seeking care in perinatal settings.

## Introduction

According to the United Nations Development Program, discrimination against sexual and gender minorities (SGMs) is “more widespread and socially accepted than virtually any other kind of discrimination in the world.”^1^ Social inclusion and non-discrimination has been identified as a social determinant of health by the World Health Organization (WHO).^2^ Such a characterisation means that those who experience discrimination and social exclusion are more likely to experience poorer health outcomes.^2^ There is growing interest within academic and policy literature globally examining how SGMs are treated across healthcare systems.^3–5^ Hsieh and Shuster^6^ have highlighted the existence of significant barriers at the systemic, institutional and patient levels, which have in turn led to poorer physical and mental health outcomes for this group when compared to heterosexual and cisgender populations.

### Sexual and gender minorities definition

Academic literature offers varied definitions for SGMs, from broad descriptions like “individuals who do not identify as heterosexual or cisgender”^7^ to exhaustive lists such as “lesbian, gay, bisexual or transgender.”^8^ Similarly, “sexual and gender minorities” also varies significantly in how it is defined in policy. Examples of these variations are visible when comparing policy from the United States’ National Institute of Health, Australian Institute of Health and Welfare and the United Nations.^1,9,10^

Mikkola^11^ notes that language in this area is evolving, with no consensus among scholars or policymakers on conceptual definitions across various sexual and gender identities. “Sexual and gender minorities” is commonly used in academic literature, often without explanation of why the term was selected or how it has been constructed. Where examined, there has been debate surrounding the appropriateness of this terminology. Some view “sexual and gender minorities” as inclusive and adaptable across various sociocultural contexts,^12^ whereas, others have suggested the term reinforces pre-existing majority norms, historic exclusion and biological determinism.^13^

While a detailed exposition of the definition of SGM is outside the scope of our study, we define the term “sexual and gender minorities” as people who sit outside the expected or accepted societal norms regarding their sexual orientation, sex characteristics, gender identity or expression. This may include individuals who identify with lesbian, gay, bisexual, transgender and other queer identities. It also includes those who do not identify with the above terms but may be gender-nonconforming, engage in same-sex relations, or prefer not to label themselves at all. Intersex people, individuals who have innate physical sex characteristics that do not align with the binary understandings of male and female bodies, are also included in this definition. SGMs are a diverse group with varied experiences, yet many face overlapping health inequities due to insensitive healthcare policies and practices.^6^

For the purposes of this study, “SGM” was selected over acronyms such as “LGBT+” as it offers a concise term which allows for greater inclusivity and sensitivity to diverse sociocultural identities.^12^ Furthermore, it captures the enormity of sexual and gender diversity across identities, bodies, orientations and behaviours while also holding space for those who do not identify as being part of any LGBTQ+ community.^1,14^ For example, despite having common shared experiences and overlapping concerns with LGBTQ+ people, there are mixed attitudes within the intersex community concerning the impact of this group being incorporated and represented through LGBTQ+ communities (e.g. AIFS use of “LGBTIQA+”^15^).

### Perinatal care definition

For the purposes of this article, perinatal care relates to the period which includes pregnancy, labour and birth and 12 months postpartum. We acknowledge this term has various definitions used by different bodies and these definitions carry policy implications. This definition was selected as it seeks to broadly capture experiences relating to accessing pregnancy-related care, including birth and postnatal care. This definition does deviate from the protocol of this study, which was created a priori and included “pre-conception.” This was changed as part of the iterative process due to reasons relating to time constraints and because both academic literature and policy most commonly do not include preconception care within the scope of perinatal care.

### Significance of the study

Calls within academic literature have been made for the development of inclusive policies in perinatal care to improve the healthcare provision of SGMs and cisgender women who are accessing perinatal care services.^16,17^ Current policies internationally and in the Australian context commonly employ a heterocisnormative lens to childbearing and make assumptions around the identity of the childbearing person, using language such as “mother” and “pregnant women.”^18–20^ Heterocisnormativity is the assumption that it is “normal” to be heterosexual and cisgender and abnormal to be nonheterosexual or noncisgender.^21^ Healthcare grounded in heterocisnormative assumptions around sex and gender has been demonstrated to cause long-term negative impacts for the health and care provision for SGMs.^6^ This in turn creates a knowledge gap for healthcare providers (HCPs) and embeds heterocisnormative assumptions and norms into practice guidelines for practitioners.

It is well documented that SGMs face stigmatisation and barriers when accessing healthcare generally.^22,23^ Healthcare policies are central to shaping provider responsibilities and accessibility to care, yet perinatal policies in Australia and internationally often overlook the needs of SGMs. Inconsistency around SGM-inclusive perinatal policies and guidelines may exacerbate poor health outcomes and care experiences for this group as practitioners do not have accurate or consistent guidance on how to meet the care needs for SGM people in the perinatal context.

In undertaking a scoping review using a thematic reflexive analysis approach of the existing international and Australian literature on childbearing SGMs accessing perinatal care, it is hoped that the results of this study may then be used to clarify the facilitators and barriers faced by SGMs accessing perinatal health services and be used to guide future research and policy on this topic.

## Materials and methods

A scoping review was selected for this study because it facilitates the mapping of all existing available research on the topic to be summarised and disseminated, providing a resource for policy makers, practitioners or service-users to review where they may not have the resources or time to scope the literature themselves.^24^ Because this is a scoping review, no quality appraisal was undertaken to ensure the greatest breadth of literature will be included.

### Study design

This scoping review followed Arksey and O’Malley’s framework and Joanna Briggs Institute (JBI) guidelines for scoping reviews.^24,25^ A scoping review protocol was developed a priori to the implementation of the search strategy. This protocol is available on Open Science Framework.

The JBI guidelines recommend using the population/participants, concept and context (PCC) framework to clarify the focus and context of the review.^25^ The PCC framework as applied to the research question for this scoping review is available in Table 1.

**Table 1.** PCC framework.

Population/participants	Childbearing sexual and gender minorities
Concept	Healthcare accessibility
Context	Perinatal care

PCC: population/participants, concept and context.

### Eligibility criteria

#### Population/participants

Research that includes childbearing SGMs who have accessed perinatal care will be eligible for inclusion. Any literature that includes experiences of SGMs in perinatal care settings is eligible for inclusion.

##### Concept

Studies that focus on the experiences of SGMs accessing perinatal care will be eligible for inclusion. For the purposes of this study, perinatal care will be defined as the period of time “from conception to 12 months after birth.”^26^

##### Context

Studies focusing on the perinatal healthcare context, whether this be in a hospital or community setting, will be eligible for inclusion.

##### Study types

Original, published, peer-reviewed, primary research of all data types (i.e. quantitative, qualitative and mixed-methods) will be eligible for inclusion. Grey literature, theoretical papers, policy documents, books, commentaries, editorials and abstract-only papers will be excluded.

##### Language

Studies written in English or translated in English will be eligible for inclusion.

##### Dates

Studies published between January 2000 and February 2024 were included for this study. Although we recognised there have been notable changes socially and culturally in relation to the rights of SGM populations globally, there have not been any recent significant policy or legislative changes impacting SGM people and their access to perinatal care, hence why we did not decide to exclude articles from a later date.

##### Exclusion criteria

All reviews, including scoping reviews, systematic reviews and literature reviews will be excluded. Studies which focus on abortion and pregnancy loss will also be excluded.

### Search strategy

This search strategy was developed in accordance with the guidelines and recommendations outlined by the JBI Evidence Synthesis and the Preferred Reporting Items for Systematic Reviews extension for Scoping Reviews (PRISMA-ScR).^25,27^ This checklist can be found in Supplementary A.

Preliminary searches were conducted across the databases Ovid Medline, Scopus, CINAHL, Embase, PsycINFO and Google Scholar to locate a “gold set” of 10 articles that were to be included in the full review. Keywords from the articles were identified and were incorporated into the search strategy where relevant, examples including “LGBT,” “perinatal” and “health access.” When testing the search strategy, if a gold set article was not retrieved, the search was revised, and relevant keywords from the articles were incorporated into the search strategy until all articles were retrieved.

The search strategy for the full literature search was developed on Ovid Medline, and subject headings and terms were converted across to CINAHL and Scopus where possible. These databases were selected as they cover international literature on healthcare, and each contained all of the gold set articles and will thus be able to retrieve a collection of papers relevant to the research topic. The Scopus database search strategy can be found in Supplementary B.

#### Study selection

Search results were exported to EndNote libraries, combined and then imported into Covidence with duplicates removed. A pilot title and abstract screening to assess for agreement between reviewers was carried out in which 10% of the articles’ titles and abstracts were screened independently by two reviewers against inclusion criteria. Inter-rater reliability of ⩾80% agreement was reached (91%), so a second pilot test was not necessary. After the pilot was completed, reviewers A and B screened 100% and 30% of articles, respectively, for both the title and abstract and full-text screening stages to ensure consistency.

#### Data extraction

Data were extracted in Covidence by two reviewers, then compiled into tabular form by one reviewer (Billie Zeta). Table 2 includes the paper ID, first author, year of publication, study design, study aims, title, sample characteristics, data collection, analysis, recruitment and the relevant findings.

**Table 2.** Extracted data.

Paper ID, first author, year, country, design, aims	Title	Sample characteristics	Data collection, analysis and recruitment	Relevant findings
1. Fischer 2021, Canada^28^ Design: qualitative Aim: to capture the unique reproduction narratives of non-binary people AFAB.	Non-binary reproduction: stories of conception, pregnancy and birth	5 non-binary individuals AFAB. Mean age 34.8 years (range 31–44 years). Majority white (4/5). Majority sexual orientation queer (4/5). All partnered, majority married (4/5). All held university degrees.	Recruitment via community contacts and social media posts In-depth, unstructured interviews lasting between 60 and 90 min via Zoom or in person. Thematic analysis.	Relevant themes: pregnancy, birth • All participants desired and sought out gender-affirming care but access was limited depending on participant’s geography and financial resources • All participants experienced misgendering, invasive questions regarding their bodies and assumptions around their families and parenting configurations and felt burdened to self-advocate, educate, explain and justify their non-binary pregnancy to HCPs, with encounters with unknowledgeable HCPs often leaving participants feeling “demoralised” and “dismissed” • Some participants experienced dysphoria due to physical changes of pregnancy or in chestfeeding their babies, however, the gendered nature of pregnancy created a lonely, isolating experience for all participants and assumptions and language of others created more feelings of gender dysphoria rather than the pregnancy itself for some • Participants used a variety of strategies demonstrating resilience: including repeatedly reminding and correcting staff on correct and appropriate language to use, some participants chose to present themselves as women to receive more compassionate, quality care
2. Gomez 2021, United States^29^ Design: qualitative Aim: to explore the experiences of TNB young adults AFAB in health care services that traditionally fall under the umbrella of “women’s” health and how health care professions, providers, and settings can create more inclusive patient-centred services	“It’s Being Compassionate, Not Making Assumptions”: transmasculine and nonbinary young adults’ experiences of “Women’s” health care settings	20 TNB individuals AFAB. Mean age 26.5 years (range 18–29). Majority identified gender identity as trans man or transgender (13/20). Range of sexual orientations. Majority identified sexual orientation as queer (13/20). Range of races/ethnicities. Majority white (12/20). Educational level not reported.	Recruitment via Facebook posts, community networks, Craigslist, and flyers in businesses, social service agencies and clinics serving LGBTQ+ clients. In-depth semi-structured interviews conducted via videoconference or in person. Thematic analysis.	Relevant themes: gendered language, disclosure of identity, assumptions of identity and embodiment, competence, humility • Gendered language in healthcare spaces served as a barrier to the provision of quality healthcare for TNB people, participants experienced a lack of opportunities to provide correct pronouns, names and gender identity on forms, electronic health records and failure of staff to identify correct terms • Insufficient clinician competence was a barrier to healthcare access and negatively impacted participants’ trust, participants experienced HCPs making incorrect assumptions about them, invasive and irrelevant questions, explicit prejudice, pejorative statements and often had information regarding their gender identity and appropriate language overlooked despite providing them providing the correct details • Many participants delayed care due to anticipated poor clinician competence due to previous negative experiences or hearing about this from other TNB people within the community, some participants travelled considerable distances to receive care from HCPs trained in transgender healthcare, as inadequate training was more common in rural or suburban areas • Participants felt secure and safe when HCPs demonstrated compassion, humility, flexibility and where it was perceived that they understood and respected participants’ identities; thoughtful, relevant, open-ended questions about needs and preferences being critical to this
3. Greenfield 2022, United Kingdom^30^ Design: mixed-methods Aim: to examine how COVID-19 service changes to perinatal care affected the experiences of LGBTQ+ parents in the UK.	LGBTQ+ new and expectant parents’ experiences of perinatal services during the UK’s first COVID-19 lockdown	1754 participants. 76 self-identified as LGBTQ+ and 1673 self-identified as cisgender and heterosexual. 5 omitted due to unavailable data for categorisation. 95.6% heterosexual, 2.9% bisexual women, 1% lesbian or gay women, 0.1% gay men. Others self-defined sexual orientation. Majority cisgender. Age not reported. Race/ethnicity not reported. Educational level not reported.	Recruitment via online advertisement and snowballing. Online survey (open and closed questions). Reflexive thematic analysis.	Relevant themes: support, recognition • During UK lockdowns, policies were designed around heterocisnormative assumptions around infant feeding that did not account for the possibility of non-gestational parents who may be lactating and feeding their babies • The care offered by healthcare professionals was seen as insufficient by many participants • LGBTQ+ parents described restrictions in ways that suggested they were targeted or amplifying existing inequalities among sexually diverse families; several gestational parents stated that non-gestational parents were denied the opportunity to fulfil their role as partners and parents • COVID lockdown restrictions caused fear and anxiety, and some participants decided to, or seriously considered, giving birth without midwifery or medical support in order to ensure they could have the presence of the people they most wanted support from
4. Hoffkling 2017, United States^31^ Design: qualitative Aim: to identify some of the needs of transgender men in the family planning process and during the peripartum period, as well as the ways they have achieved empowerment, opportunities for supporting their further empowerment and priorities for further investigation through a systematic qualitative study	From erasure to opportunity: a qualitative study of the experiences of transgender men around pregnancy and recommendations for providers	10 self-identified transgender men. This article did not report demographic information. The only information available was gathered below from the inclusion criteria: • 18 or older. • Self-identified as male before pregnancy. • Pregnant within the last 10 years. • Able to complete the study in English.	Recruitment via pool of prior participants of an online convenience sampled survey of transmasculine individuals who had given birth. Online semi-structured interviews conducted online via video remote-conferencing programme, two conducted via audio only. Grounded theory.	Relevant themes: diversity, structuring barriers, erasure, and transphobia, positive experiences with HCPs, anticipatory guidance throughout the family planning process, optimism • Participants connected with supportive communities, made decisions to limit interactions with health services (including one participant electing for a home birth) to help them to avoid transphobia and overcome challenges they faced • The need for gender affirmation varied; for some participants gender-affirming care was critical, others were minimally bothered by being misgendered • Participants felt that the system conveyed that their lives and identities could not exist within the system; participants reported that IT systems did not allow for male pregnancies, there was a lack of information available due to both inadequate scientific research and HCP training, and physical spaces, decoration and educational materials felt like they were catering only to cisgender women • Participants had many experiences around a lack of cultural competence, including: misgendering, making incorrect assumptions, ignoring intake forms that specified parents’ gender, presuming a patient has/should have a particular kind of relationship with their body, performing unnecessary physical exams, tokenising comments, asking invasive and voyeuristic questions and overt transphobia • Participants reported that the absence of any models of pregnant transgender men and the discourse around the pregnant man was unintelligible to others and was disempowering for participants • Participants generally cared more about being accepted and respected rather than providers being able to answer all their biomedical questions; HCPs naming and normalising gender and differentiating between what they personally did not know and what science did not know while jointly discussing uncertainty and evaluating risk with their patient were reported as positive experiences and helped to establish trust
5. Jackson 2022, United States^32^ Design: qualitative Aims: to explore LGBTQI+ parental experiences regarding their interactions with healthcare professionals as a resource for feeding options during prenatal-to-neonatal period	What are LGBTQ+ parental experiences of healthcare support and decision-making regarding infant feeding options? A grounded theory study	21 LGBTQI+ parents of infants who were born in a hospital or birthing centre and were <1 year of age Age ranged from 26 to >46. Range of sexual orientations. Majority homosexual (14/21) All in legally recognised civil union or married. Majority highest level of education bachelor’s degree or graduate and/or doctorate degree (18/21) Range of annual household incomes. Ethnicity not reported.	Recruitment via social media. Demographic survey and semi-structured interviews in-person or via videoconference lasting 60–90 min. Grounded theory	Relevant themes: education, continuity of care, parental engagement, open communication • Participants who attended prenatal classes described them as “mommy-centric” and assumed the partner was “dad” in most cases, creating feelings of alienation • HCPs limited conversations around breastfeeding to confirming that the gestational parent would breastfeed and few discussions promoted dialogue around alternative feeding options; one couple used informal milk sharing without HCP input due to fear of being reprimanded by the health team. • Participants felt HCPs were generally respectful of their gender identity and families valued continuity of care when accessing lactation services postpartum and after hospital discharge, especially when they faced breastfeeding challenges • Most participants relied on community connections or online resources to identity lactation specialists or find information to guide decisions relating to feeding rather than rely on services offered through hospital or physician offices as they perceived HCPs to be lacking in knowledge to support LGBTQI+ parents on alternative feeding methods
6. Klittmark 2023, Sweden^33^ Design: qualitative Aims: to explore birth and non-birth parents’ experiences of care from HCPs during births where obstetrical or neonatal complications arose and to generate knowledge that can contribute to better care and treatment of LBTQ people in birthing care	When complications arise during birth: LBTQ people’s experiences of care	22 LBTQ parents who experienced birth complications. Mean age 34.2 years (ranged 28–40). Majority gender identity cisgender women (20/22). All participants married or cohabitating with co-parent at time of child’s birth. Range of sexual identities. Majority lesbian. Majority university degree or university student. Race/ethnicity not reported.	Recruitment via social media and snowballing. Semi-structured individual interviews, lasting between 46 and 120 min. Thematic analysis.	Relevant themes: invalidated as an LBTQ family, disrespectful treatment during birth, lack of information and support • A majority of participants experienced a lack of availability from HCPs resulting in a lack of necessary information and emotional support during and after their complicated births, leaving participants vulnerable in highly stressful situations; many participants interpreted lack of information and support to be caused by lack of resources in birthing care and the stressful working environment for HCPs which made them forget or be indifferent to participants’ needs • Most participants experienced disrespectful treatment and violations of their bodily or personal integrity from HCPs which became a part of their traumatic birth experiences, experiences included painful forced examinations done without consent or against their expressed will, negative, punitive or threatening comments directed at them, exotification, misgendering and heterocisnormative assumptions • All participants wanted HCPs to see and validate them as LBTQ people and in LBTQ families and validation as LBTQ family was often more highly valued than other care needs
7. Lancombe-Duncan 2022, United States^34^ Design: qualitative Aims: To explore the experiences of multi-level stigma and resilience among LGBTQ+ people in the context of conception, pregnancy and loss.	Minority stress theory applied to conception, pregnancy, and pregnancy loss: A qualitative study examining LGBTQ+ people’s experiences	17 LGBTQ+ people who had experienced pregnancy loss or in an intimate partnership in which pregnancy loss in the last 2 years. Mean age 34.4 (ranged 29–40) Majority cisgender women. Range of sexual orientations. Majority queer. Range of race/ethnicities. Majority white. Majority married. Majority graduate degree. Majority living in urban geography.	Recruitment via social media and word of mouth. Screening and demographic survey and semi-structured individual interviews ranging 25–70 min. Thematic analysis.	Relevant themes: Interpersonal stigma, interpersonal stigma, structural stigma, individual resilience, relational resilience, collective resilience • Heteronormative assumptions and exclusion of LGBTQ+ people were embedded in systems, including through a lack of options on intake forms; there was particularly a lack of representation of LGBTQ+ people of colour in information materials and healthcare settings • Participants reported that HCPs made heterocisnormative assumptions and insensitive and hurtful comments and judged participants for their reproductive choices, holding LGBTQ+ people to different standards compared to heterosexual/cisgender people and making participants feel unwelcome in health and social service settings • Participants demonstrated resilience in a number of ways, including: asking for what they needed, knowing their limits and setting boundaries, building relational resilience through meaningful relationships with HCPs, members of online LGBTQ+ support groups, and through developing their own LGBTQ+ friendly resources and materials such as blogs • Some participants reported positive reactions from HCPs that participants found meaningful and helped build resilience
8. McNair 2008, Australia^35^ Design: qualitative Aims: Explore how lesbian parents negotiate the health care system	Lesbian parents negotiating the health care system in Australia	61 individuals from 20 lesbian-parented families. 36 lesbian parents, 20 children, 3 grandparents and 2 donors/fathers. Ages ranged 29–62 years. Range of ethnicities. Majority from Anglo-Australian backgrounds. Educational level not reported. “Wide range of economic backgrounds” Majority living in inner metropolitan location.	Recruitment purposive and snowball sampling via lesbian community and professional networks. Note: only in the state of Victoria Families interviewed in one single in-depth unstructured interview lasting 1.5–5 h, sometimes punctuated by a meal break. Grounded theory.	Relevant themes: experiences with the health care system and providers, disclosure of lesbian sexuality within the health care system • No participants described overt homophobia, but many described heteronormative assumptions; some participants experienced HCPs not immediately embracing their sexuality but becoming more comfortable over time • Lack of recognition in the healthcare system and on data collection forms of lesbian-parented families; highlighted to families they did not “fit” as they would have hoped • Participants used different approaches around disclosure of their sexuality: private, proud and passive; these strategies were flexible and adjusted according to context and risks • Some participants described positive experiences which involved being accepted as normal, feeling respected, comfortable, or safe with their provider
9. Parker 2023, New Zealand^36^ Design: qualitative Aims: to respond to two main questions. First, how does cisnormativity operate in perinatal care settings? Second, what impacts does cisnormativity have on trans people accessing perinatal care?	“It’s Total Erasure”: trans and nonbinary people’s experiences of cisnormativity within perinatal care services in Aotearoa New Zealand	20 trans and nonbinary people. Range of gender identities. Half nonbinary (10/20). Ages ranged 18–40. Range of ethnicities. Majority Pākehā/New Zealand European (13/20). Educational level not reported.	Recruitment via social media and word of mouth. Semi-structured interviews held in-person or via videoconferencing. Reflexive thematic analysis.	Relevant themes: cisnormative physical environments and structures, cisnormative language and interactions, impacts of cisnormativity • HCPs did not have gender-inclusive models to draw from, leaving some HCPs not knowing how to respond to disclosure of gender with almost all participants experiencing misgendering and some participants experiencing having their gender erased, questioned, challenged or denied. Participants had to weight up risks around disclosure or self-advocacy for receiving gender-affirming care, which could be stressful or risky, and detract from their capacity to focus on their pregnancy health needs • Although some individual HCPs made efforts to be inclusive, these were identified as isolated efforts that were not apparent in the wider infrastructure of perinatal care spaces; participants described seeking out signs of trans inclusion but that recognition gender diversity was rarely visible within perinatal settings, participants attributed erasure of gender diversity to systems not being “designed” to recognise or accommodate gender diversity as seen through the gendered organisation and labelling of space, lack of gender-neutral spaces, and the lack of opportunities to self-determine language • A range of strategies were used in self-advocacy or self-protection, including asking HCPs to use correct name and pronouns, and sending information on gender-affirming care to HCPs before appointments, performing femininity, some participants elected to birth out of hospital to avoid risking exposure to an unsafe and non-affirming perinatal care environment • Some participants experienced visceral reactions to being misgendered and having to self-advocate to receive gender-affirming care compounded stresses of navigating experiences of discomfort about their body changes
10. Ril 2024, Brazil^37^ Design: qualitative Aims: to understand experiences of double motherhood during antenatal, childbirth and postpartum health care.	You only have one mother!: Institutional violence in experiences of double motherhood in healthcare	9 self-identified cisgender lesbian or bisexual women. Majority lesbian (6/9). Age range of 21–49. Majority same-sex relationships at the time of participation (8/9). “Self-declared skin colour, by criteria of Brazil’s official bureau of statistics”: majority white. Majority undergraduate student or higher educational level. Various places of residence.	Recruitment via hashtag #duplamaternidade (#doublemotherhood) on Instagram. Individual open interviews, online and asynchronous, and an asynchronous online focus group Thematic content analysis.	Relevant themes: cisheteronormativity and its impact on double motherhood experiences, institutional violence in health services: from curiosity to LGBTQIA+phobia • Participants discussed how monitoring instruments such as the pregnant woman’s card upheld heterocisnormative assumptions by listing only options as “mother” and “father,” erasing the lived experience and identities that do not align with the normative model • Participants experienced institutional violence including heterocisnormative assumptions that exotified or erased the role of the non-pregnant mother; in one case, the non-pregnant mother was excluded from the birth of her child, and the gestational parent was deprived of her partner during birth to prioritise the learning opportunity of students
11. Salden 2023, Germany^38^ Design: quantitative Aims: To examine whether and to what extent the experiences of different gender identities and sex characteristics differ in obstetric care	Cisnormativity, erasure, and discrimination: how do trans, non-binary and intersex persons experience obstetric cagermanre compared to endosex cisgender individuals in Germany?	1431 individuals. 864 heterosexual, cisgender, endosex participants 438 cisgender queer endosex people 88 non-binary endosex people 14 endosex transgender men 7 intersex people Race/ethnicity not reported Education not reported	Dissemination via social media and email sent within queer communities to queer parenting groups, queer associations, organisations and activists and to mainstream organisations working on reproduction, birth and parenting for non-queer pregnant participants. Online survey. Exploratory factor analysis using principal component methods and varimax rotation.	Relevant themes: Interactional level: Experiences in interactions with medical staff, institutional Level: Obstetric care institutions, structural level: access to information and appropriate materials, results in relation to intersex people • Trans men and non-binary people had worse experiences compared to non-queer people and to a lesser extent, than cis queer people; they experienced more discrimination and violence giving birth in hospitals, felt less welcome in hospitals and received less competent and respectful care; some experienced misgendering by medical staff • A majority of respondents who gave birth in hospital did not know whether there were corresponding complaint offices in the hospital; some participants communicated with friends, midwives or even sued doctors to seek outside support of the hospital • 45% of cis queer, 46% of non-binary and 33% of trans men reported that medical personnel during pregnancy and postpartum assumed heterosexuality “for the most part” • Almost all information was heteronormative, cisnormative and endonormative creating barriers for queer, trans and intersex people accessing information • Intersex people felt the worst informed by doctors and midwives and had the most difficulty obtaining appropriate information; some could not become pregnant at all due to childhood surgery performed without their consent and intersex participants called for an urgent end to medically non-emergency surgeries on intersex infants
12. Santos 2024, Brazil^39^ Design: qualitative Aims: to analyse the culturally constructed meanings regarding the bond with healthcare services and professionals by lesbian and bisexual women who experienced dual motherhood	Lesbian and bisexual couples experiencing dual motherhood: (dis)encounters in the provision of healthcare	10 self-identified cisgender lesbian or bisexual women Age range 30–39. Race/ethnicity not reported. Range of educational levels. Majority higher education or higher (i.e. post-graduation). Majority married (9/10).	Recruited via snowballing from online group on the topic of double motherhood. Online unstructured interviews lasting 51–92 min. Geertz’ Interpretative Theory of Culture.	Relevant themes: building links with health services and professionals • Participants experienced non-gestational parents being excluded from appointments, forcing gestational parents to advocate for themselves to be recognised by their family and challenge heteronormative assumptions made by HCPs, with some participants experiencing HCPs suggesting there is a “real” (gestational) mother and a “fake” (non-gestational) mother, with non-gestational mothers being equated as “the man” in the relationship which they found offensive
13. Searle 2017, Canada^40^ Design: qualitative Aims: to broaden current understandings of trauma, examine structural marginalisation within perinatal care relationships to provide insights into the impact of dominant models of care on queer birthing women.	Accessing new understandings of trauma-informed care with queer birthing women in a rural context	13 LGBQP2S (lesbian, gay, bisexual, queer, pansexual and two spirit) women Ages ranged 18–42 years. Range of races/ethnicities. Majority white (10/13). “The spectrum of socio-economic status ranged from working poor to upper class with the majority of participants identified as working class” “Range of educational backgrounds from GED to PhD preparation” All participants lived in a rural location.	Recruitment via advertisements in clinics, hospitals, community bulletin boards, social media and queer events and word of mouth. Semi-structured interviews in-person or via telephone lasting 60–90 min. “Analysis guided by feminist and queer phenomenology”	Relevant themes: withholding birthing spaces for queer: lack of validation for difference, potentiating difference, healing at the centre: validation for creating collaborative and equitable care • Queerness was not always discussed or acknowledged by HCPs, continuing cycle of queer invisibility and structural trauma that left participants feeling uncertain about safety and forcing participants to challenge cisheteronormativity, which risked retraumatisation • Participants experienced a lack of proactive disruption of heterocisnormative assumptions and lack of HCPs recognising the potential for difference, making participants feel disempowered and “at the mercy of the system,” forcing them to come out in resistance to HCPs enforcing hetercocisnormativity
14. van Amesfoort 2023, the Netherlands^41^ Design: qualitative Aims: to explore the needs and barriers of transgender men in family planning, pregnancy, childbirth, puerperium and perinatal care	The barriers and needs of transgender men in pregnancy and childbirth: a qualitative interview study	4 transgender men, 1 nonbinary individual Ages ranged 23–35 Race/ethnicity not recorded. Educational level not recorded.	Participants were identified by clinical team at the Center of Expertise on Gender Dysphoria and invited to participate. Semi-structured interviews conducted in-person or via videoconferencing lasting 40–60 min. Grounded theory.	Relevant themes: from reproductive intent to conception, pregnancy, puerperium and parenthood, perinatal care • Participants noted that most pregnancy and childbirth facilities address cisgender women and their male partners and reported discomfort in physical spaces such as waiting rooms as they felt others were staring at them • HCPs used wrong pronouns, misgendering and lacked adequate awareness of the specific needs of participants; one participant was denied care by a HCP because they did not feel they had the knowledge to support them • Participants leaned on support from family, friends and partners and sought out information and community groups online to find materials relevant to them • Participants who received specialist care for TGI people described this as a positive experience as participants did not have to explain their identity, however not all participants were able to access this specialised service due to barriers created by distance • Becoming a gestational parent was a significant milestone for all participants experiencing pride and joy, with a majority of participants describing feeling like a father to their child without feeling incongruence with their gender identity • Physical changes resulting from pregnancy caused dysphoria for some participants and physical examinations involving exposed genitalia were a source of dread with some participants expressing concern that their genitalia would be exotified; some participants opted for caesareans due to natural birth triggering dysphoria
15. Wingo 2018, United States^42^ Design: qualitative Aims: to explore LGBTQ FAAB individuals’ priorities and experiences with reproductive health care	Reproductive health care priorities and barriers to effective care for LGBTQ people assigned female at birth: a qualitative study	39 LGBTQ people assigned female at birth Range of sexual orientations. Majority queer. Range of gender identities. Majority female. Mean age 29.9 (ranged 18–44). Range of races/ethnicities. Majority white. Education ranged from less than high school to postgraduate degree. Majority college graduate or higher.	Recruitment through community-based social networks, including LGBTQ listservs, professional networks, postings at local LGBTQ organisations, and Craigslist and snowballing In-depth interviews conducted in-person or via phone or videoconferencing. Thematic analysis.	Relevant themes: Fertility and women’s care focus, LGBTQ erasure and competency, discriminatory comments and care, impact on reproductive care-seeking behaviour • Heterocisnormativity through inflexible and unspecific intake forms and reproductive health centres named “women’s health centres” created obstacles to care for individuals by producing discomfort and anxiety for participants, or creating barriers in communication • Participants perceived a lack of knowledge in relation to providing accurate medical guidance for LGBTQ+ participants, notably the effects of testosterone for transgender patients • Participants experienced homophobic and transphobic remarks from HCPs, which negatively impacted their desire to seek future care – these negative experiences could also be shared across LGBTQ+ communities and discourage care-seeking behaviour across the community due to anticipated discrimination
16. Gonzales 2019, United States^43^ Design: quantitative Aims: To compare an array of health outcomes between pregnant sexual minority women and pregnant heterosexual women	Health and access to care among reproductive-age women by sexual orientation and pregnancy status	67728 participants. 3901 cisgender sexual minority women and 63827 cisgender heterosexual women. Ages ranged 18–44. Range of race/ethnicities. Majority white. The following data is for pregnant sexual minority women participants only: 42.5% married or living with a partner. Majority educational level was high school graduates or lower.	Recruitment via community-based social networks and snowballing. Second wave of targeted recruitment was conducted to increase diversity via social media and Craigslist. Cross-sectional telephone survey. Statistical analysis.	Data not categorised by themes • Substantial disparities in health care access, mental health, physical health, and health risks for sexual minorities compared with heterosexuals, with pregnant sexual minority women more likely to report frequent mental distress, a depression diagnosis, poor physical health days, activity limitations, at least one chronic health condition and unmet medical care needs owing to cost compared with pregnant heterosexual women

AFAB: assigned female at birth; TNB: transgender and nonbinary; LBTQ: lesbian, bisexual, transgender and queer; HCP: healthcare provider.

#### Data analysis

Quality appraisal was not undertaken as is common practice for scoping reviews so as not to reduce the quantity of studies to be included within the review.^24^ Peters et al.^25^ state “for many scoping reviews, the analysis of the extracted data should not involve anything more than basic descriptive analysis.” However, this scoping review will be analysed using Braun and Clarke’s^44^ six phases of reflexive thematic analysis: familiarisation, coding, generating initial themes, developing and reviewing themes, refining, defining and naming themes and writing up.

This was selected over a basic descriptive analysis approach as we feel that a reflexive thematic approach is better equipped to provide a richer and more insightful understanding of the phenomenological experiences of childbearing SGMs accessing perinatal care. This is an interpretive approach that utilises and values the subjectivity of the researcher and analyses the data reflexively to draw out rich, nuanced understandings of knowledge.^44^

Furthermore, the reflexive thematic analysis approach allows us to understand the broader social context in which SGMs experience perinatal care in the context of healthcare services. For these reasons, Braun and Clarke’s thematic reflexive approach was used to analyse the data in this scoping review.^44^

#### Presentation of results

Results are presented in two broad sections as per Peters et al.^25^ The first includes information broadly describing the search strategy results and key information; the second provides the thematic analysis of the results as relevant to the research question.

## Results

### Overview

**Figure 1.**
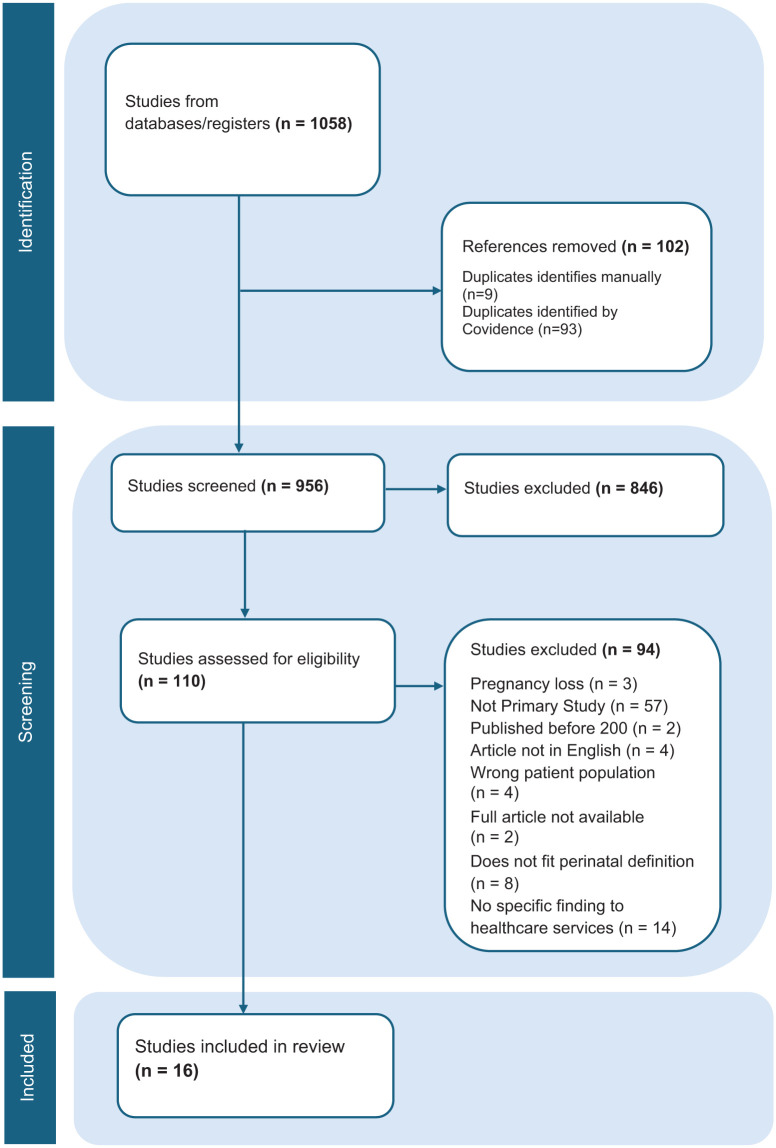
PRISMA-ScR flow diagram. PRISMA-ScR: Preferred Reporting Items for Systematic Reviews extension for Scoping Reviews.

The database searches identified 1058 articles, 102 were removed as duplicates. Of the remaining 956 studies, 846 articles were removed for ineligibility through the title and abstract screening. Of the 97 full-text articles assessed, 16 met eligibility requirements. Reasons for exclusion can be found in Figure 1.

The 16 articles for inclusion included 13 qualitative papers using interviews and/or focus group methods, 2 quantitative papers using surveys and 1 mixed-methods study. The earliest research evidence was from 2008,^8^ with all other articles published after 2017. Six articles originated from the USA, two articles came from Brazil and the United Kingdom, and the remaining six articles were from Sweden, Germany, the Netherlands, Canada, New Zealand and Australia. Studies from Brazil and Australia focused exclusively on lesbian and/or bisexual women’s experiences, whereas other countries examined experiences from more diverse SGM identities.

Data collection strategies included surveys, unstructured and semi-structured interviews and a focus group. Qualitative articles used thematic analysis, grounded theory, Geertz’^45^ interpretive theory of culture or feminist and queer phenomenology. Meanwhile, quantitative studies used statistical analysis or exploratory factor analysis. Sample sizes ranged from 5 to 61 for qualitative interviews, 1431 to 67,728 for quantitative or mixed-method surveys. Six participants were included in the focus group from Study #10.

Recruitment strategies predominantly used social media, community organisations and snowball sampling (1; 2; 3; 5; 6; 7; 8; 9; 10; 11; 12; 14; 15; 16). Participants varied in age, gender and sexuality, although a majority were white, highly educated and partnered. Ethnicity and education data were collected in inconsistent categories across studies. Only one study included intersex people (11). The extracted data can be found in Table 2.

### Reflexive thematic analysis

Five themes relevant to the experiences of childbearing SGMs accessing perinatal care were identified: structural stigma, interpersonal stigma, affirmation of identity, resilience and positive experiences.

#### Structural stigma

Across all global contexts, the theme of structural stigma was overwhelmingly covered. Institutional policies and societal level biases created major barriers for SGM individuals accessing perinatal care. Forms and data systems were frequently reported to use restrictive or unclear language, which failed to reflect the realities of sexual and gender diverse populations (1; 2; 4; 7; 8; 9; 15). These practices which embedded heterocisnormativity into perinatal care “institutionally erased” (7) and “violate[d] identities that fail[ed] to align with the normative model.”^10^

Physical spaces, classes and materials were described as reported to be designed for cisgender heterosexual women (1; 4; 5; 6; 7; 8; 9; 11; 14; 15; 16). This “mommy-centric” (5) approach to care left SGM individuals feeling uncomfortable and alienated when trying to access these supports (1; 4; 5; 6; 7; 8; 9; 11; 14; 15; 16). Key information, such as the impact of testosterone on pregnancy, was commonly identified as lacking (4; 14; 15), which prompted SGM individuals to seek out and rely on community sources, which could provide medically unreliable information and advice (5; 7; 14).

The sole article that included intersex participants found that they were the least informed and faced the greatest difficulty accessing relevant care (11). Some intersex individuals could not become pregnant due to non-consensual or unnecessary surgeries performed on them as infants, with participants calling for an end to non-emergency surgeries on intersex infants (11).

Quantitative literature revealed significant disparities in perinatal care for SGM people (11; 16). Sexual minority women reported worse physical and mental health outcomes compared to heterosexual women (16), while trans men and non-binary people received less competent care and more experiences of discrimination and violence compared to queer cisgender people and even more so compared to non-queer cisgender people (11).

#### Interpersonal stigma

Interpersonal stigma from perinatal HCPs was reported frequently in the literature. Experiences of misgendering and making heterocisnormative assumptions were widespread (1; 2; 4; 6; 7; 8; 9; 10; 11; 13; 14; 15). These experiences forced SGMs to choose between invisibility or self-advocacy, with both options carrying risk and emotional burdens to themselves and/or their families (1; 9; 13). Even when corrected, HCPs could repeat the same mistakes in subsequent interactions (2; 4; 9).

The literature frequently reported that SGMs experienced HCPs lacking in training and cultural competence in SGM-specific care, leading to some avoiding or delaying care (1; 2; 4; 5; 6; 7; 8; 9; 10; 11; 12; 14; 15). This manifested in a variety of ways, including: calling SGM individuals by the incorrect name (2; 4; 11), discussing gender like it is sexual orientation (4), using incorrect titles or pronouns (2; 4; 6; 9; 11), suggesting pregnancy is incompatible with masculine gender identities (4; 14) and not recognising the parenthood of non-gestational parents (3; 5; 8; 10; 12). Two studies reported transgender individuals being denied care by providers due to a lack of knowledge of their care needs or negative attitudes towards their gender identity (4; 14).

SGMs commonly experienced invasive or irrelevant questions and comments from HCPs about their identities, bodies and experiences (1; 2; 4; 6; 7; 10; 11), with participants describing these experiences as “objectifying” (4) and “dehumanis[ing]” (2). Some SGM individuals reported unnecessary or non-consensual physical exams, violating their body integrity (4; 6). Overt prejudice, including pathologisation of identity and threats of child removal, further eroded trust and caused significant distress in their own care (2; 4; 9; 11; 15).

#### Affirmation of identity

Although it was common for SGM individuals to value identity affirmation (3; 4; 6; 8; 9;13), the need for affirmation varied, ranging from minimal concern resulting from misgendering or a lack of recognition, while others experienced “visceral” distress (4; 8; 9). Trans and nonbinary participants reported dysphoria from pregnancy-related body changes or being feminised by others due to their pregnancy (1; 4; 14).

Recognition of their SGM identity was essential for some individuals, sometimes being valued more than other care needs (4; 6). However, for others, controlling disclosure was essential in ensuring they felt safe in their care as it allowed them to hide their identity to avoid discrimination, protect themselves and their families and to receive more compassionate care (1; 2; 8; 9). One study highlighted how this was not always a choice for people who are forced to attend “women’s” clinics, with one trans man stating, “if you see anyone in that clinic with a beard and a deeper voice, he’s trans, no stealth.”^2^

#### Resilience

SGM people showed resilience by using strategies like self-advocacy to navigate and resist heterocisnormativity in perinatal care. This included correcting language, setting boundaries and educating providers (1; 6; 7; 8; 9). These efforts were, however, reported as burdensome and adding additional stress to their pregnancy (1; 6; 8; 9).

SGM individuals commonly developed relational resilience through meaningful relationships with HCPs, family, friends, or other supportive communities (2; 3; 4; 5; 7; 8; 14; 15). SGM-specific communities were reported as helpful in locating and accessing affirming information and care (2; 4; 5; 7; 14; 15).

When facing or anticipating prejudice and discrimination, SGM individuals employed strategies such as managing the choice of disclosure (1; 4; 8; 9), limiting interactions with HCPs (4; 8), seeking care elsewhere (2; 4; 8), choosing home-births (3; 4; 9; 11), pursuing legal action (11), or some trans and nonbinary people, presenting as women to access higher quality care (1; 4; 9).

#### Positive experiences

Incidence of positive experiences ranged from being rare or exceptional to common (1; 4; 6; 14). Positive experiences were marked by feelings of safety, trust, respect and were free from heterocisnormative assumptions (4; 8; 9; 14). SGM individuals valued HCPs’ awareness of language, including correct use of names, pronouns and inclusive recognition of diverse family configurations and bodies (1; 2; 4; 5; 6; 7; 8; 9; 10; 11; 12; 13; 14; 15).

Medical and cultural competence fostered safety for SGM people (1; 2). Access to gender-affirming practitioners reduced the need for self-explanation and led to more tailored care for transgender and non-binary people (1; 14). Creative solutions, like private appointments or coordinating with external services, also helped in building trust (14).

SGM individuals valued HCPs who asked thoughtful questions, acknowledged uncertainty and collaborated on informed decisions (2;4). Providers who educated themselves and showed humility, openness and willingness to learn eased the burden on SGMs in educating their practitioners (2; 4; 9; 14). One study highlighted the importance of HCPs “differentiat[ing] between ‘I don’t know’ and ‘science doesn’t know.’”^4^ Continuity of care also assisted in building trust and reduced the need for repeated disclosures (5; 6).

## Discussion

This scoping review aimed to collate, synthesise and map the existing literature on the experiences of childbearing SGMs accessing perinatal care and to explore the facilitators and barriers experienced in this healthcare context. This section will discuss implications of the findings of this research, areas for future research and limitations of this study.

One challenge of this study was capturing the diversity and nuanced experiences of specific subgroups of SGMs, which was reflected across the broader and included literature. The included studies lacked discussion around the language chosen to define their populations. Some papers (reference and context) offered brief definitions, while others provided no definition at all. The US Institute of Medicine^46^ highlights the need for researchers to provide operational definitions of sexual orientation and gender identity to better allow for comparison and combination of data across multiple studies. Definitions for studies focusing on sexual minority groups did not specify whether sexual orientation was defined by behaviour, attraction or identity (8; 10; 12; 16). Studies focusing on transgender or nonbinary people were more specific, outlining definitions centred around identity (1; 2; 4; 9; 14). The remaining studies used umbrella terms such as “LGBT” and did not discuss the limitations of using such broad labels, such as the potential failure to capture nuances and differences across different communities (3; 5; 6; 7; 11; 13; 15). There was a general lack of discussion around the political implications of categorising populations or the potential exclusions this creates. The implication is a broader one for future research, pointing to a need for clarification and acknowledgement of the rationale and limitations of language choice when investigating SGM communities.

There was also a notable lack of sociodemographic data collected in much of the literature. For instance, 43.8% of articles did not report educational level or race or ethnicity. Where this data was collected, all studies reported majority white or Anglo participants and had university-level education or higher. Other demographic categories such as religion, (dis)ability, indigeneity and geography (i.e. rural status) were rarely collected. Consequently, there is an overrepresentation of people from white, high socioeconomic backgrounds and limits intersectional analysis of perinatal care access. Any policy or interventions derived from the current literature may therefore fail to address the specific needs of underrepresented groups. Future research should report more comprehensive sociodemographic data and increase diversity within participant populations to broaden learning, allow for deeper analysis of intersectional identities and increased generalisability of results.

Only one study (11) included in this review involved intersex participants, highlighting a significant gap in our understanding of the experiences of childbearing intersex people accessing perinatal care. This may in part be due to common clinical practices across the world performing medically unnecessary surgeries on intersex infants, children and adolescents that can lead to permanent infertility, systematically removing the ability to become pregnant from many intersex people.^47^ Nevertheless, this demonstrates an endonormative bias within academic literature that fails to examine intersex experiences in perinatal care. The lack of data of any distinct experiences or healthcare needs of intersex people would act as a barrier to the development of adequate and inclusive guidelines that ensure competent and supportive care for intersex individuals.

This review found that although literature on understanding the lived experiences of SGM people in perinatal care is limited, it is rapidly increasing, with most articles being published within the last 5 years. The literature included in this review highlighted several barriers emerging from heterocisnormativity being embedded within perinatal healthcare, manifesting through the structural and interpersonal discrimination against SGMs. The exclusionary design of perinatal care institutions create structural discrimination and social exclusion which uphold heterocisnormative ideals around the traditional family, alienating and isolating SGM people and their families. These barriers were shown to create distrust and deter SGM people from seeking care due to the risk of discrimination, with quantitative studies demonstrating poorer health outcomes when compared to general populations. Existing research indicates stigma may not only impact childbearing people, but also their infants, and may result in long-term negative health and economic complications for healthcare systems and individuals.^48,49^ These barriers may discourage SGM individuals from envisioning pregnancy and parenthood as viable options to begin with.^50^

This study also found that affirmation and recognition of identity was commonly identified as an important aspect of care for SGM parents. Although this study was centred around capturing the experiences of childbearing individuals, previous research has found that symptoms of anxiety and depression increase during the perinatal period for non-gestational parents, and may be compounded with feelings of disentitlement and abandonment by social and medical systems.^51,52^ This points to a need to emphasise the full inclusion of SGM families in perinatal care settings and extend care to non-gestational parents to support SGM families as individuals and as a whole to feel prepared, supported and engaged throughout the perinatal period.

Furthermore, the prevalence of interpersonal stigma that was perpetuated by HCPs indicates a need for increased education in SGM care needs, sensitivity training and other mechanisms to ensure accountability and shifts towards a more inclusive and culturally competent workplace culture in perinatal care settings. Overall, these findings are consistent with wider background literature that has found SGM individuals experience discrimination and barriers to care in health settings^6^ and represent a wider need for inclusive and effective policies that address the health and care needs of SGM individuals accessing perinatal care. The World Professional Association for Transgender Health’s (WPATH) Standards of Care Version 8 publication contains recommendations for healthcare professionals providing care to transgender, gender-diverse and intersex people, which may be learned from and adapted into the perinatal context.^53^

This review identified several facilitators which supported SGM people to access perinatal care, including their use of creative strategies to navigate challenging heterocisnormative systems. While these approaches fostered strength, they also carried an emotional toll, as SGMs often had to protect themselves from exclusionary environments. Many turned to online health information when providers lacked inclusivity or knowledge. However, limited health or web literacy can lead to misunderstandings,^54^ highlighting the need for accurate, SGM-inclusive resources. Further research into online health information access and quality is warranted. Overall, this highlights the importance and need for inclusive policies and practice guidelines that address the root of stigma and prevent the burden from falling solely on SGMs. This was supported by the findings around positive experiences in the literature which showed that affirming, responsive care from HCPs helped reduce anxiety and fostered trust in the perinatal healthcare system.

Ultimately, the findings of this study demonstrate a need for further research into more diverse populations and the development and implementation of inclusive policies and practice guidelines across the perinatal health system to facilitate SGM peoples’ access to care. The findings of this study could be used to inform the evaluation of existing policies and procedures within perinatal care services and support the establishment of inclusive practice guidelines that will facilitate access of SGM people in perinatal care.

### Limitations

This study had some limitations. Due to time and resource constraints, only 30% of articles were independently screened by two reviewers. A pilot screening of 10% was therefore conducted beforehand, achieving 91% agreement to ensure a high level of inter-rater reliability (⩾80%). Additionally, excluding non-primary studies and non-English texts may have omitted valuable non-Eurocentric or secondary insights. However, focusing on primary, peer-reviewed research was a deliberate choice to ensure rigour, support policy relevance and identify key knowledge gaps for future research.

## Conclusion

This study identified several themes across the international literature on the experiences of childbearing SGMs accessing perinatal care. Barriers such as structural and interpersonal stigma, which embedded heterocisnormativity at both macro and micro levels, led to feelings of discomfort, alienation, vulnerability and fear for SGM people. Facilitators in accessing perinatal care for SGM people included receiving compassionate, affirming care and practitioners with relevant medical and cultural competence to address the needs of SGM patients, or openness and humility to educating themselves on these topics. Resiliency skills such as self-advocacy and adaptability also supported SGM people to navigate their care, however, this carried its own emotional burdens.

This study identified gaps in the existing literature, including limited attention to the gendered, political and ethical implications of defining SGMs in research, a lack of inclusive perinatal policy development and a scarcity of recent Australian research. It also highlighted future research priorities, including exploring more diverse and intersecting identities, intersex experiences and the quality of online health information for SGMs. Addressing these gaps could help reduce barriers and inform inclusive policies and care practices for the diverse communities encompassed by the SGM umbrella.

## Supplemental Material

sj-docx-1-whe-10.1177_17455057261444891 – Supplemental material for The experiences of sexual and gender minorities accessing perinatal care: A scoping review Supplemental material, sj-docx-1-whe-10.1177_17455057261444891 for The experiences of sexual and gender minorities accessing perinatal care: A scoping review by Billie Zeta and Tejaswini Patil in Women's Health

sj-docx-2-whe-10.1177_17455057261444891 – Supplemental material for The experiences of sexual and gender minorities accessing perinatal care: A scoping review Supplemental material, sj-docx-2-whe-10.1177_17455057261444891 for The experiences of sexual and gender minorities accessing perinatal care: A scoping review by Billie Zeta and Tejaswini Patil in Women's Health
