# Supplementary material for: The experiences of sexual and gender minorities accessing perinatal care: A scoping review
Source: Womens Health (Lond). 2026 May 10;22:17455057261444891. doi: 10.1177/17455057261444891 (PMC13167375; doi:10.1177/17455057261444891)
Supplement: sj-docx-2-whe-10.1177_17455057261444891 – Supplemental material for The experiences of sexual and gender minorities accessing perinatal care: A scoping review [file sj-docx-2-whe-10.1177_17455057261444891.docx]

Supplementary B. Scopus Search Search Strategy

| DATABASE and Search Strategy | Limits |
| --- | --- |
| **Scopus**  ( ( ( TITLE ( transgender ) OR ABS ( transgender ) ) ) OR ( ( TITLE ( queer ) OR ABS ( queer ) ) ) OR ( ( TITLE ( trans-men ) OR ABS ( trans-men ) ) ) OR ( ( TITLE ( trans-masculine ) OR ABS ( trans-masculine ) ) ) OR ( ( TITLE ( transmasculine ) OR ABS ( transmasculine ) ) ) OR ( ( TITLE ( nonbinary ) OR ABS ( nonbinary ) ) ) OR ( ( TITLE ( non-binary ) OR ABS ( non-binary ) ) ) OR ( ( TITLE ( gender-diverse ) OR ABS ( gender-diverse ) ) ) OR ( ( TITLE ( bisexual ) OR ABS ( bisexual ) ) ) OR ( ( TITLE ( pansexual ) OR ABS ( pansexual ) ) ) OR ( ( TITLE ( intersex ) OR ABS ( intersex ) ) ) OR ( ( TITLE ( gay ) OR ABS ( gay ) ) ) OR ( ( TITLE ( homosexual ) OR ABS ( homosexual ) ) ) OR ( ( TITLE ( lesbian ) OR ABS ( lesbian ) ) ) OR ( ( TITLE ( "women who have sex with women" ) OR ABS ( "women who have sex with women" ) ) ) OR ( ( TITLE ( "assigned female at birth" ) OR ABS ( "assigned female at birth" ) ) ) OR ( ( TITLE ( "female assigned at birth" ) OR ABS ( "female assigned at birth" ) ) ) OR ( ( TITLE ( gender-fluid ) OR ABS ( gender-fluid ) ) ) OR ( ( TITLE ( genderfluid ) OR ABS ( genderfluid ) ) ) OR ( ( TITLE ( lgbt* ) OR ABS ( lgbt* ) ) ) ) AND ( ( ( TITLE ( ( health W/3 ( "enabl*" OR "facilitat*" OR "access*" OR "barrier*" ) ) ) OR ABS ( ( health W/3 ( "enabl*" OR "facilitat*" OR "access*" OR "barrier*" ) ) ) ) ) OR ( ( TITLE ( ( care W/3 ( deficienc* ) ) ) OR ABS ( ( care W/3 ( deficienc* ) ) ) ) ) OR ( ( TITLE ( inclusi* ) OR ABS ( inclusi* ) ) ) OR ( ( TITLE ( barrier* ) OR ABS ( barrier* ) ) ) OR ( ( TITLE ( challenge* ) OR ABS ( challenge* ) ) ) ) AND ( ( ( TITLE ( perinatal ) OR ABS ( perinatal ) ) ) OR ( ( TITLE ( prenatal ) OR ABS ( prenatal ) ) ) OR ( ( TITLE ( antenatal ) OR ABS ( antenatal ) ) ) OR ( ( TITLE ( postnatal ) OR ABS ( postnatal ) ) ) OR ( ( TITLE ( pregnancy ) OR ABS ( pregnancy ) ) ) OR ( ( TITLE ( birth ) OR ABS ( birth ) ) ) OR ( ( TITLE ( ( care W/2 ( "pregnancy" OR "preconception" OR "maternity" ) ) ) OR ABS ( ( care W/2 ( "pregnancy" OR "preconception" OR "maternity" ) ) ) ) ) ) |  |
